# Promoting Recovery in Mental Illness: The Perspectives of Patients, Caregivers, and Community Members in Dar es Salaam, Tanzania

**DOI:** 10.1155/2020/3607414

**Published:** 2020-06-06

**Authors:** Masunga K. Iseselo, Joel Seme Ambikile

**Affiliations:** Department of Clinical Nursing, Muhimbili University of Health & Allied Sciences, P.O. Box 65001, Dar es Salaam, Tanzania

## Abstract

**Background:**

Promoting mental health and care in the community setting leads to the recovery of patients with mental illness. Although recovery in mental health is a complex phenomenon, caregivers and community members have important roles to play in the recovery process for patients with mental illness. Little is documented on how recovery is promoted in the community setting. This study explored the experience of patients, caregivers, and community members on how recovery can be realized in a patient with severe mental illness in Dar es Salaam.

**Methods:**

We conducted four focus group discussions (FGDs): two with caregivers and the other two with community members. Also, six in-depth interviews were held with patients with mental illness. Participants were purposively selected based on the type of information needed. Both FGD and in-depth interviews were digitally recorded and transcribed. Qualitative content analysis was used to analyze data. *Findings*. Four themes emerged from this study, which include promoting patients' participation in household activities, improving patients' support system, promoting patients' self-care management, and providing safety and protection among patients with mental illness. However, financial, psychological, and establishing care and support centers and professional supports emerged as subthemes from patients' support system.

**Conclusion:**

Caregivers and community members are significant stakeholders for promoting recovery for people with mental illness. The current study reveals that patients' involvement in home activities, promoting self-care management, improving patients' support systems, and providing safety and protection are important factors that promote recovery for people with mental illness. Advocating mental health awareness for caregivers and community members will bridge the gap to enhance the recovery for people with mental illness. Further research is needed in this area to explore the health care providers' perspectives on the recovery process of mental illness in the hospital setting.

## 1. Introduction

Globally, mental illness causes tremendous morbidity, mortality, and impairment. It is estimated that mental illness and other psychological disorders account for about 10% of the disability-adjusted life years (DALYs) [1]. The majority of people with mental illness remains untreated or seeks traditional treatment particularly in low-income countries [2, 3]. Although some programs to provide services for mental illness at primary care level have been developed in low-income countries, these are generally limited because of poor integration of mental health into primary health care and inadequate resources [4–6].

In Tanzania, community-based mental health services are inadequate at all levels of the health care delivery system. The first contact with mental health care providers begins to happen mainly at the district level where professional support is provided to facilitate recovery [7]. Several challenges such as lack of social support, disruption of family functioning, stigma, and discrimination affect the mental health care system in Tanzania [8]. Inadequate clinical care and lack/low availability of support services may lead to a poor recovery process among people with mental illness.

Recovery takes numerous meanings depending on the situation in which it is explained, and the word may create confusion among caregivers, patients, and professionals as well as policymakers and researchers. Research on recovery among various mental health populations and on effective strategies and interventions to promote recovery is still in its infancy [9]. The concepts of recovery of patients with mental illness are about staying in control of their life rather than the subtle state of returning to the premorbid level of functioning [10]. The approach which does not focus on full symptom resolution but emphasizes resilience and control over problems and life has been named as the recovery model [11–13]. Also, personal and clinical recovery had been described as distinct entities that play a part in the patient's recovery [14]. In these perspectives, personal recovery focuses on living a satisfying, hopeful, and contributing life even with limitations caused by the illness. Clinical recovery focuses on sustained remission and restoration of functioning and does not change across patients with mental illness [15].

People with severe mental illness present with numerous difficulties in their social functioning. Patients with mental disorders such as bipolar disorders, schizophrenia, schizoaffective, and depressive disorders are at increased risk of delayed recovery [16, 17]. The reasons for this are reported to be a lack of energy and motivation to engage in community activities, as a result of the mental disorder, side effects of psychotropic medications, and prolonged hospitalization [18]. Research shows that people with mental illness tend to fail in meeting socialization needs because most of them avoid social relations and spend most of the time alone, thus living a sedentary lifestyle [19]. In the home or community environment, most caregivers do not get time to be with their patients to help them meet the required needs or involving them in daily activities. It is also revealed that schizophrenic patients, especially those who are chronically ill, have much worse social support networks in terms of quantity and quality of care [20]. The situation may be exacerbated when patients with mental illness receive support less frequently from close family members, friends, and other relatives. The absence of social support is usually associated with psychiatric symptoms, poor perceptions of general health, and reduced ability for integration in the community [17].

Recovery from a mental illness is facilitated by many factors including social support, patient participation in daily activities, self-care, and the employment needs of the patient [21]. It is documented that participation in home-care services promotes good functioning in social situations and protects against frequent hospitalizations [18, 22]. Involving a person with mental illness in daily activities plays a key role in the measurement of functional health which is the component of the recovery process [23]. A systematic review has shown that regular physical activity performed in the home environment is widely recognized as a protective factor against the overall burden of disease [24] and hence promotes recovery from mental illness. Moreover, participation in recreational groups and socially supported physical activity is shown to reduce stress, anxiety, and depression, as part of the recovery process. Evaluations of some programs related to the care of patients with mental illness found that physical activity is increased when the social environment is supportive [19, 22]. In recent years, there has been an increase in awareness about the role of caregivers in the long-term management of psychiatric patients, and there is a growing body of literature on the importance of meeting mental health needs that facilitate recovery [21, 25]. Unmet needs of people with mental illness are a serious public health problem; hence, changing attitudes and behaviors towards mental illness promotes recovery [26]. It is necessary to provide interventions for people with severe mental illness within the spheres of the family and community.

There is a lack of information regarding the recovery process when the patient with severe mental illness is cared for at home setting in Tanzania. The current study explored the views and experiences of patients, caregivers, and the community members on the recovery process of a person with severe mental illness cared at home in Dar es Salaam.

## 2. Material and Methods

### 2.1. Study Design

This was a qualitative descriptive study aimed at exploring the views and experiences of caregivers, patients, and community members on promoting recovery from mental illness in the community setting.

### 2.2. Study Setting

The study was conducted in Temeke district, one of the five districts of Dar es Salaam. It has a surface area of 729 km^2^. In 2019, Dar es Salaam had a population distribution of 6,701,650 with a growth rate of 5.23% [27]. The residents of this area are the mixture of indigenous and immigrant people from other regions especially southern Tanzania. The extended family pattern is predominant than the nuclear family in most areas of Dar es Salaam [28]. Administratively, Temeke district is divided into three divisions and 24 wards. The district has four hospitals and more than a hundred dispensaries and health centers. Most patients get outpatient mental health services at Temeke Hospital, which is one of the two government hospitals. Temeke Municipal Hospital plays both roles of receiving patients with mental illness from the community and providing follow-up care. There is no inpatient mental health service at the hospital. Patients who need hospitalization are referred to Muhimbili National Hospital.

### 2.3. Study Participants

The study participants included mentally ill patients, caregivers, and community members. The inclusion criteria were patients having a chronic mental illness as defined by Bachrach [29] and who were mentally stable. To determine their mental stability, psychiatrists clinically assessed the mental status of the potential participants during their clinic visits at Temeke Hospital. Caregivers were the primary ones who had stayed with a mentally ill patient for at least six months. This was the minimum amount of time for them to experience the mental health needs that facilitate recovery for the patient as reported by Mavundla and colleagues [30]. The community members must have had lived in the area for more than 3 years and reported to be exposed to or interacted with a person with mental illness in his or her life.

### 2.4. Sampling Procedure

Convenient sampling was used to get six patients during their follow up clinic at Temeke Hospital. Patients with known conditions such as schizophrenia, major depression, and bipolar disorders but mentally stable (with minimal/no psychotic symptoms) were recruited in the study. The attending psychiatrist confirmed the diagnosis depicted by patients' medical records. Additionally, we recruited caregivers who accompanied the patient during the follow-up clinic at the hospital. In most cases, one caregiver accompanied a patient. We asked only the primary caregiver when more than one relative escorted the patient. However, some patients visited the clinic alone, and in this case, they were asked to provide a contact address or phone number of their primary caregiver to be contacted for an interview. Caregivers who agreed to participate were asked to come to the hospital for discussion. We also recruited thirteen community members from the local setting. With collaboration with the local leader, we identify the community members who were eligible and willing to participate in the study and set the appropriate time for the discussion. Specifically, the community members were neighbours of a patient with mental illness and people with experience of living with a mentally ill patient. Although mental health providers are important in the recovery process, we did not include them as this study intended to explore the recovery process of patients in the community and home environment.

### 2.5. Data Collection

Four focus group discussions (FGDs) and six in-depth interviews were conducted during data collection. FGDs were conducted among caregivers and community members and six in-depth interviews with patients attending the mental health clinic at the facility.

#### 2.5.1. In-Depth Interview for Patients

We interviewed the patients in a quiet room with minimal interruption from other patients and staff because it was a bit far from the clinic site but within the hospital premises. During patient interviews, the contents covered included the family and community support received, community perception towards the patients, and patient involvement in various daily activities. A semistructured interview guide was used to collect data. The interview guide was constructed and pretested as described by McIntosh and Morse [31]. The following were the questions that guided the interview:

1. Do you think you receive adequate support from family and community members as far as your mental illness is concerned? Why?
2. What types of support do you receive at home and in the community?
3. What challenges do you face in getting this support?
4. How do people in the community view you as far as your mental illness is concerned?

These questions were followed by the specific probing questions to get more details or clarification of the responses provided by participants. Data saturation was reached at 6 interviews, the degree to which new data repeated was expressed in previous data as guided by Hennink et al. [32]. The duration of the interview lasted from 30-45 minutes.

#### 2.5.2. FGDs with Caregivers

The FGDs were conducted to get experience from caregivers' perspectives on patients' support received and patients' involvement in different household and community activities. The following semistructured questions guided the group discussion with caregivers:

1. What do you think are the needs for the recovery of a mentally ill patient at home?
2. What are your views about how these needs are met?
3. What support have you been providing to facilitate the recovery of your patients at home? What are the challenges?
4. What would be the best way to facilitate recovery for your patients?

Each question was followed by several probing questions as well.

Female and male participants were interviewed separately to facilitate the free expression of ideas.

#### 2.5.3. FGDs with Community Members

The discussions were conducted at the local cell leader's office in the evening (after working hours) to avoid disturbance during the discussion. Participants actively engaged in the group discussion. The questions that were asked included the following:

1. What do you think are the needs for recovery of a patient with mental illness in your community?
2. How are these needs met to facilitate recovery of mental illness?
3. What would be the best way to assist patients towards recovery?

The FGDs for caregivers and community members were conducted with care to maintain homogeneity. Two homogeneous groups comprising 6 men and 7 women were involved in the discussion with community members and the other two with caregivers, comprising 7 males and 9 women. The degree of data saturation was reached at a total of 4 FGDs. That is, we stopped recruiting new FGDs when new data repeated what was expressed in the previous data as defined by Hennink et al. [33]. Each FGD lasted between 40 and 60 minutes.

Both in-depth interviews and FGDs were conducted in Swahili, a native language for all participants, and were digitally audio-recorded. Data that was not easily captured by the audio recorder such as nonverbal cues and environmental events were recorded in the notebook. In the case of FGDs, the researcher was leading the discussion and keeping the conversations flowing, and a research assistant was recording the interviews and taking notes.

### 2.6. Data Analysis

After data transcription, authors began reading and rereading the transcribed interviews separately with an open mind to obtain a general impression. Interview notes and ideas were written, and the transcripts were read several times so that the researchers could be immersed in the data and allow different ideas raised by the participants to be captured. Additionally, field notes were correlated with the transcripts to enrich the texts.

**Figure 1:**
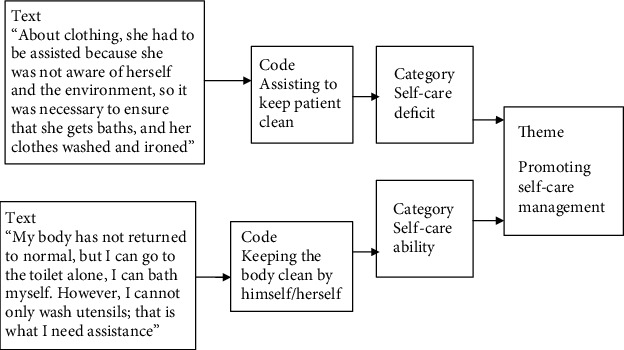
The coding process and formation of themes.

Then, the transcripts were entered into computer NVivo 10 software. This tool is designed to help in organizing, analyzing, and getting insight into unstructured qualitative data [34]. The coded text was filtered and placed in similar contents that formed a tree node. The identified content of the text was entered into memos, which were used to summarize the patterns of the condensed meaning. Memos were eventually manually organized into pattern subcategories and categories. To identify the source of data in the memo and in the text, identification (ID) numbers of informants were used, so that the source could not be easily tracked and accessed. Different categories, condensed meaning units, or codes were compared for underlying meaning and relationship at the interpretation level that formed the themes. Some excerpts of relationships between these categories that formed the themes are shown in Figure 1.

During the process of analysis, the authors had to discuss meanings arising from the analysis outputs, core meanings, category, and themes, according to the objectives of the study. Some themes were omitted for lack of relevance such as patient needs. We also held meetings with caregivers and community members to discuss and agreed with the themes and subthemes identified. This increased the credibility of our study. The themes were described supported by artificial quotations, maintaining as far as possible the meaning of original terminology that was used by the informants.

### 2.7. Ethical Clearance

The ethical approval of the study was obtained from the Institutional Review Board of the Muhimbili University of Health and Allied Sciences, and permission to conduct the study was sought from the district medical officer and local leaders. Participants were informed that their involvement in the study would provide the opportunity to gain information that would be useful in improving mental health services. Confidentiality was guaranteed by using numbers to identify informants and that the audio-recorded transcripts and written notes containing the participants' information would be kept well in a safe place and would be destroyed after completion of the study. Written informed consent was obtained from all participants before the interview sessions. For patients with mental illness, informed consent was also obtained first from their primary caregivers.

## 3. Findings

### 3.1. Characteristics of Informants

The study comprised three groups of participants; patients, caregivers, and community members. Patients are those who were mentally stable and were able to communicate properly (see Table 1). Caregivers were those who were key carers and lived with the patients for more than 6 months. Community members involved in the study were residents of that area.

### 3.2. Perspectives of Patients, Caregivers, and Community Members in Promoting Recovery of Mental Illness

The participants' views and perspectives were grouped into four themes. These are patients' involvement in the home activities, improving patients' support systems, promoting patients' self-care management, and providing safety and protection as described in the following sections.

#### 3.2.1. Patients' Involvement in the Home Activities

Caregivers and patients expressed different views and experiences about patients' involvement in daily activities. Some showed negative attitudes towards patients' participation in the home activities and others had positive attitudes. Participants with positive attitudes believed that occupying patients with household activities could relieve the symptoms of mental illness. They emphasized that caregivers were responsible to involve the patient as much as possible. However, they expressed that patients were not able to do any work at home, as expressed by one of the caregivers:

*“There is another problem; he* (patient) *cannot work. He tells you that he is mentally ill... I was very disappointed with the patient that he cannot do anything. I do not know why it was that way”* (female caregiver for a patient with schizophrenia).

Depending on the severity of the patient's condition, some caregivers expressed a different experience; their patients had at least the ability to do some basic activities at home depending on their capability and the enabling environment. In other words, the ability to do home activities was not uniform among the patients as reported by the caregivers. Some needed more activities compared to when they were not ill or when there were no symptoms as expressed by caregivers:

“*If the patient is in that condition* (mental illness), *he cannot forget to bathe, eat, and even other household activities. There is an increased need to do activities compared to when the patient is well”* (female caregiver for a patient with bipolar disorders).

She also added:

“*Sometimes he can be sitting alone until you remind him what he is supposed to do, although this is not a serious issue that affects him a lot”* (female caregiver for a patient with bipolar disorders).

On the other hand, patients expressed their ability to do home activities depending on the level of their cognitive ability. Those who were students were able to attend class sessions and clean their home compounds. Others verbalized that they were able to be involved in home activities such as cooking, though with some assistance and supervision as described by patients:

*“I can do household activities and attend some class session as well as making cleanliness of the compound and collaborate with my relatives in other activities”* (male bipolar patient).

*“I fulfill my responsibility such as cooking because I am not too ill to need assistance, I can do myself as usual”* (female schizophrenia patient).

Various reasons affecting the involvement of the patient in daily activities were given including the effects of medication, male dominance, and using the illness as an excuse. Caregivers described that their patients could not be involved in home activities due to medication effects and that affected their performance of household activities as expressed by one of the caregivers:

*“… sometimes before they get medication, they are usually active, but after getting the medication they slowdown in their activities*” (male caregiver for a patient with schizophrenia).

Patients' inability to work or do home activities was sometimes perceived as a pretense or malingering. Caregivers were concerned that some patients were pretending and using the illness as an excuse for not being involved in any activities at home as stated by a participant:

“*If you ask him, he could tell, ‘don't you see that I am sick?' So even if you were angry you have to refrain and endure his actions”* (female caregiver for a patient with a bipolar patient).

#### 3.2.2. Promoting Self-Care Management

Patients gave different views on how they participated in various self-care activities. Most of them were able to meet their basic needs to some extent. Normal body hygiene is usually difficult to meet in severe mental illness. However, some patients reported meeting these needs especially when debilitating symptoms disappear as stated by patients:

“*My body has not returned to normal, but I can go to the toilet alone, I can bath myself. However, I cannot only wash utensils; that is what I need assistance”* (female schizophrenic patient).

Caregivers' views on patients' self-care were similar to what patients said. They said in the active phase of symptoms patient cannot do anything unless the condition was improved. So, they ensured that the patient was clean and provided needed support like attending to the toilet as described:

*“He was defiling his clothes with stool, if I see that I take his clothes and wash them, I bath the patient, but now he has improved that he can eat and go to the toilet by himself”* (female caregiver for a patient with schizophrenia).

Another participant commented on the importance of patient cleanliness:

*“About clothing, she had to be assisted because she was not aware of herself and the environment, so it was necessary to ensure that she gets baths, and her clothes washed and ironed”* (female caregiver for patient with major depression).

#### 3.2.3. Improving Patients' Support System

Caregivers and patients expressed various views and experiences on the improvement of the support system to facilitate patient recovery. In particular, financial, emotional, love and affection, housing and treatment camps, and professional supports were mentioned as the important contributors to patient recovery if provided.

*(1) Financial Support*. Most patients received financial supports from their relatives and other family members. It helped them to buy medications and food and meet other basic needs as expressed by a patient:

*“I do get support; my family and relatives help me with all kinds of assistance. The family is with me and has not neglected me because of my mental illness. I am grateful for the financial assistance that they provide for purchasing medication, food and other several personal needs”* (male patient with bipolar).

Some patients were able to do some small business to gain some money for buying their items such as medication that improves patients' symptoms as stated by patients with schizophrenia and depression: others received financial support from significant others:

“*I do small business that enables me to get money for buying medication. Because of this medication, my condition now improving”* (male patient with schizophrenia).

*“My husband buys everything I need. He is very careful with the financial issues when I get any problem; my husband helps me”* (female patient with major depression).

*(2) Psychological Support*. The emotional support was mentioned as an important factor for patient recovery. It was obtained from different sources within the community and at home. Caregivers verbalized that the presence of someone who could give emotional and psychological support, including counseling, was important for patient recovery. One of the caregivers expressed this:

“*In most cases, my patients get emotional support, i.e. your presence because; sometimes they pass through different problems. I do not know what is in their mind, you need to be there to give counseling and the like. He* (the patient) *only needs to talk with you, to be with you, because sometimes he cannot handle a lot of stress, pressure, etc. Doing this can facilitate patient improvement”* (male caregiver for a patient with depression).

The need for love and affection for people with mental illness was expressed by caregivers as one of the important aspects that can facilitate recovery. They commented that most patients suffer a lot from lack of care and love from their close relatives and significant others. Knowing the importance of love, most caregivers reported offering love and warmth to their patient, and in so doing, patients' symptoms were improved as explained by the caregiver:

“*I think the most important the patient needs to get is love and care. Because, if you love him; he feels loved and cared for”* (female caregiver for a patient with schizophrenia).

“*The service which I give to my mom is love. That is if you show the love she calms down from emotional stress. Now she is doing fine”* (female caregiver for her patient with depression).

Likewise, community members reiterated the importance of love and warm care to people with mental disorders. They said patients need assistance and lovely responses that may make them feel needed and welcomed as stated as follows:

*“I think all communities need to show love and caring attitudes to people affected with mental illness. Because of the high percentages of people with mental illness lack lovely care. For example, he* (patient*) can be having a problem and comes to you for help, if you cannot help you need to respond politely, if you respond harshly, he can change suddenly. So, love is very crucial for these people so that they can improve their condition”* (female community member, age 40 years).

*(3) Establishing Caring and Support Centers*. Most community members expressed the need for residential treatment camps where people with mental disorders could be gathered and treated. They suggested that the government should establish these camps to allow patients to get food and professional treatment. They emphasized that the camps would help to reduce caregivers' burden and reduce the number of homeless patients with mental illness as stated as follows:

“*I would like to ask the government to build residential camps for this type of patients so that they* (patients) *could get their services in these camps, and they would be getting good nutrition and treatment”* (female community member, age 29 years).

Another participant added:

*“Basically, the government should build residential camps like orphanage centers. The patient could be placed there so that doctors could be attending and treating them”* (male community member, age 36 years).

Community members further stated that residential treatment centers could be a solution to people with mental illness scattered in the streets. Getting patients into these camps would help to carry out a proper assessment to determine their needs rather than leaving them in the street as expressed in the following:

*“To eliminate this problem of having people with mental illness scattered in the community, they should be placed in some special areas for more assessment to know their needs and problem”* (male community member, age 40 years).

*(4) Promoting Professional Support*. Professional treatment was also mentioned as an important aspect of a support system that could facilitate the recovery of people with mental illness. It was considered crucial to consult mental health care providers for treatment and other mental health services. This would help most patients roaming in the street as exemplified by a community member:

*“…we as community members, when we have such patients, we need to take them to the hospital so that they can be treated through medications, and that will help much rather than doing nothing”*(male community member, age 28).

Other participants emphasized the importance of professional support as this could help to rule out other physical problems such as cerebral malaria and other problems that can mimic symptoms of mental illness:

“*…when we see such a person, we have to take him to the hospital because at the hospital, there are various investigations that will let them* (professionals) *know the source of the problems. Sometimes, you think he is mentally ill; you take him to the traditional healer, oh! You find that he has cerebral malaria. Or you can say that he has a mental illness but, to the contrary, he has taken drugs and other substance that affects the mind”* (female community member, age 40 years).

#### 3.2.4. Providing Safety and Protection

Another important aspect that participants expressed was the safety and protection of patients. Community members were concerned that some patients, especially females, were at increased risk of violence and sexual assaults. They expressed that rape was common among female mentally ill patients, and some of them get pregnant. This would affect the mother's recovery and the newborn baby exposed to the unfriendly environment:

*“Because the way they* (patients) *are left to wander in the street, the female patients with severe mental illness can be impregnated. The baby born will be at risk of getting an unfriendly environment. That means two peoples become affected, the baby and the mother. This is a can delay recovery for this patient”* (female community member, age 35 years).

Participants also explained that patients should be protected from injury and psychological trauma that could be caused by people in the streets. Mentally ill patients as human beings need their rights to be protected. This can increase the wellbeing and facilitate recovery as expressed by one of the participants:

“*It would be better people like these to be protected so that they do not get injured by people in the community because if it becomes known that they are not normal, they can be in danger anyhow and this is a barrier for mental health improvement”* (male community member, age 44 years).

Participants reported a lack of awareness of mental disorders as a contributing factor to the improper handling of patients in the community. People with mental illness are sometimes mistreated and beaten by those who do not know about mental illness. Caregivers expressed concern for their patients, especially on informing the community members to avoid mistreating people with mental disorders. Instead of beating them, they should be taking care of them by any possible means:

*“I try to send some messages to the youths. Also, I do walk around in the community to notify people that ‘whenever you find him* (the patient) *doing anything bad, arrest and tie him with a rope or piece of cloth. Find transport to bring him home. I will pay the cost”* (male caregiver for a patient with epileptic psychosis).

Others explained the importance of close observation, especially for patients roaming in the street. Caregivers had to take responsibility to create awareness among community members so that they take care of patients whenever they see them in the streets.

“*…also, close observation is needed for the mentally ill patient who wanders in the streets. I decided to pass through his friends and inform them that he* (the patient) *is not normal, be careful with him”* (male caregiver for a patient with schizophrenia).

## 4. Discussion

This is the first study of the kind to be conducted in Tanzania. It highlights how the community and home environment facilitates the recovery process of mentally ill patients. The main findings from this study show that most patients with mental illness do not have their needs met appropriately to facilitate recovery. The involvement of the patients in various social activities, either at home or in the community, is not adequately practiced since some caregivers and community members are not aware of how people with mental disorders could be assisted to meet their needs. Reinforcing self-care activities is an important factor to be considered by caregivers and significant others if self-care deficit is observed. Improving the support systems by the community members, caregivers, and other stakeholders is important for facilitating patient recovery. Moreover, safety and protection underscore the importance of the recovery process of a person with mental illness. This study has illuminated the important aspects that need to be implemented to achieve maximum recovery of people with mental illness in the community and home environment.

This study shows that caregivers and patients have mixed experiences of patients' involvement in various home activities. The effects of psychotropic medication that affects mostly psychomotor skills can be attributed to the views that patients are unfit to be involved in household activities. However, when patients' adherence to psychotropic medication is good, symptoms of mental illness are reduced, and social participation or involvement in different activities is improved. On the other hand, some psychotropic medications decrease the psychomotor skills of patients due to different side effects like extrapyramidal side effects (EPS) and sedation. These side effects slow down the patient's psychomotor movements and hence unfit for social and household participation. This concept is contrasted by a study in the USA which reported the involvement of persons with mental disorders in social activities to facilitate recovery [21] and another one in Sweden [35] which suggested that social activities are important and can be used in diagnostic groups of patients. Although the medication was mentioned as one of the contributing factors that limited the patient's participation in the household activities, most caregivers did not know how the illness affected the patients, thus failing to fulfill normal activities. Harvey and Trassnig reported that cognitive disability contributes to loss of performance in social activities for chronic schizophrenic patients [36], and this explains their low degree of participation. Caregivers should design social activities based on specific disabilities portrayed by patients.

Although some caregivers were satisfied by patient involvement in some simple activities at home, research from other countries shows that patients should be taught or assisted to perform structured daily activities, which should be planned based on their ability [37]. In this study, the reasons for not engaging in home activities were feigning and using illness as an excuse. There is no documented data showing that the patient, as an exemption from fulfilling daily home activities, can falsify psychiatric symptoms. However, a study done in Iran to determine what symptoms are imitated by a malingerer in forensic institutions did not show any correlation between the true symptoms and the imitated symptoms among malingerers [38]. This gives the impression that psychiatric symptoms cause cognitive disability that impairs the patient's participation in activities of daily living.

This study reports that patients acknowledged their ability to fulfill basic needs, specifically to care for themselves. This may depend on the patient's condition and the stage of the recovery process. In other studies, patients with mental illness usually failed to meet their needs due to the effects of symptoms [39, 40] and medication side effects [41]. Caregivers are supposed to ensure that the important needs of patients are met to facilitate recovery from mental illness. Grooming and clothing needs were the most expressed by both the patients and caregivers in this study. In the hospital environment, nurses may facilitate grooming when providing nursing care. However, after discharge, this role is assumed by the caregiver of the patient. As reported in this study, caregivers are usually committed and devoted to ensuring that patients are as clean as possible. Similar findings had been reported more than three decades in California where mentally ill patients in the community were taught to keep themselves clean [42].

Improving patients' support systems is an important factor that should be taken into consideration when caring for patients with mental illness in the community and home setting. Financial support is required, especially for purchasing patients' medications and transport to attend follow-up clinics and medication refills. Our study revealed that patients get financial support from significant others. However, a study in the same setting reported financial constraints among caregivers and patients as a contributing factor for not accessing mental health facilities [8]. Importantly, people with severe mental illness need to be supported financially as they may obtain a reliable source of income for existence. A study in the US revealed that the diagnosis of severe and persistent mental illness often leads to reduced work activity, hence lowering of earnings [43].

Emotional support is a prerequisite for the improvement of symptoms and facilitates recovery among persons with mental illness [22]. The provision of emotional support is regarded as a gold standard for promoting the recovery of a patient with mental illness. Love and affection are among the elements of psychological support. Our study shows that caregivers and community members can render emotional support to patients to promote their wellbeing. In other community settings, people seem to be disengaged from an emotionally unstable person, especially if the person has mental disorders [44]. Moreover, the current study indicated that people with mental illness receive love and affection from their close relatives and community members around them. This situation favours quick recovery for their conditions. In their study describing love and mental health, Maryam and Bhatia reported that love can work for everyone, but people who suffer from depression or other mental ailments will need more than love to lead a normal and healthy life [45]. Contrary to this, people with mental disorders are usually stigmatized and discriminated from social activities, which increases the social distance between them and the community around them, leading to the lack of love and affection [46].

Furthermore, our findings report housing and treatment camp support as an important parameter for reducing homelessness among mentally ill persons. This indicates that patients with mental illness can get their needs and adequate treatment rather than being cared at home by relatives. The efficacy of these camps in facilitating recovery to mentally ill patients in low- and middle-income countries is not well documented. However, a study in Ghana reported the interest of biomedical providers to partner with prayer camps in assisting the recovery of persons with mental illness [47]. Likewise, Underwood et al. in the US reported promising results to residential treatment centers that incorporate family and community support systems [48]. The findings in our study suggest that residential treatment is the alternative strategies to reduce the caring burden borne by the caregivers and the community as a whole. This can facilitate recovery of mental illness if well planned and implemented.

Although professional support as revealed in our study is crucial for investigation and stabilization of patients' symptoms, little is usually done by the family and community members to take the patient to the hospital due to beliefs attached to the cause of mental illness and influence of traditional healers. The current findings stressed the importance of taking people with mental health problems to the hospital for investigation. However, contrasting findings from a previous study in Ilala district in Dar es Salaam reported that patients sought help from traditional healers first before they consult the professional care providers [49], which is similar to other studies reported elsewhere in Africa [50, 51]. Despite this, our study might be presenting the prevailing sociocultural beliefs on mental illness that need to be considered in professional care.

Regarding patients with mental disorders being insecure, more effort is required by different stakeholders to protect them from humiliating behavior such as rape and getting pregnant, injury, and any other threat that jeopardizes human nature. It is also essential to protect the public from any danger caused by the patients. However, there is no adequate data that show to what extent the rights of people with mental illness have been violated in Tanzania. A research study in India highlighted that the human rights of people with mental illness are not protected and recommended the legal framework to protect them [52]. In the United Kingdom, patients with severe mental illness are at substantially increased risk of domestic and sexual violence, with a relative excess of family violence and adverse health impact following victimization [53]. This calls for all people around to take action in ensuring that the patient's safety and protection are realized. It is also worthwhile to note that raising awareness in the community can improve the safety and security of people with mental illness. The mass media, especially radio and television, help educate people about mental illness [54]. Mass media can instill awareness among people leading to reduced incidence of rapes and related cases that stagnates the recovery process, particularly in female patients.

Although this study has reported substantial information that is not much studied in Tanzania, it was limited by some factors such as not carrying out in-depth interviews with caregivers and noninvolvement of mental health care providers. This could elicit more information about patients' needs and their experiences than it was captured in patients. However, the information obtained gives insights on how the community and home environment can be used to plan and foster the recovery of a person with mental illness in a similar environment.

## 5. Conclusion and Implication

Caregivers and community members are the most important stakeholders for promoting recovery for people with mental illness. The current study reveals that patients' involvement in the home activities, promoting self-care management, improving patients' support systems, and providing safety and protection are important factors that promote recovery for people with mental illness. This study implies that advocating for mental health awareness among caregivers and community members will enhance recovery for mentally ill patients. Our study suggests higher authorities such as national, regional, and district mental health coordinators to collaboratively work with the care providers to improve the patients' support systems, which is crucial for the recovery process for the person with mental illness. Further research is needed in this area to explore the health care providers' perspectives on the recovery process of mental illness in the hospital setting.

## Figures and Tables

**Table 1:** Characteristics of informants.

Characteristics	Frequencies		
	Patients (*N* = 6)	Caregivers (*N* = 16) (6 for the patient with schizophrenia; 7 for bipolar patient; 3 for the depressive patient)	Community members (*N* = 13)
*Age (years)*			
20-29	2	3	3
30-39	1	7	5
40-49	2	5	4
50-59	1	1	1
60 and above	0	0	0
*Sex*			
Male	2	7	7
Female	4	9	6
*Marital status*			
Married	1	9	8
Separated	2	1	0
Divorced	0	0	1
Single	3	2	2
Cohabiting	0	4	2
*Occupation*			
Student	1	0	0
Employed	0	2	2
Retired	1	0	3
Self-employed	2	11	6
Not employed	2	3	2
*Diagnosis*			
Bipolar disorders	2		
Schizophrenia	3		
Major depression	1		

## Data Availability

The data used to support the findings of this study are available from the corresponding author upon request.
